# Dipsticks and point-of-care Microscopy in Urinary Tract Infections in primary care: Results of the MicUTI pilot cluster randomised controlled trial

**DOI:** 10.1371/journal.pone.0332390

**Published:** 2025-10-08

**Authors:** Peter K. Kurotschka, Martin J. Koch, Eva Bucher, Adolfo Figueiras, Jochen Gensichen, Alexander Hapfelmeier, Alastair D. Hay, Christian Kretzschmann, Oliver Kurzai, Thiên-Trí Lâm, Kathrin Lasher, Orietta Massidda, Linda Sanftenberg, Guido Schmiemann, Antonius Schneider, Anne Simmenroth, Stefanie Stark, Lisette Warkentin, Mark H. Ebell, Ildikó Gágyor, on behalf of the Bavarian Practice-Based Research Network (BayFoNet)

**Affiliations:** 1 Department of General Practice, University Hospital Würzburg, Würzburg, Germany; 2 Department of Preventive Medicine and Public Health, Universidade de Santiago de Compostela, Santiago de Compostela, Spain; 3 Institute of General Practice and Family Medicine, University Hospital, LMU Munich, Munich, Germany; 4 Institute of General Practice and Health Services Research, TUM School of Medicine and Health, Technical University of Munich, Munich, Germany; 5 Institute of AI and Informatics in Medicine, TUM School of Medicine and Health, Technical University of Munich, Munich, Germany; 6 Academic Unit of Primary Health Care, NIHR National School for Primary Care Research, Department of Community Based Medicine, University of Bristol, Bristol, United Kingdom; 7 Institute for Hygiene and Microbiology, University of Würzburg, Würzburg, Germany; 8 Department of Cellular, Computational and Integrative Biology, Interdepartmental Center of Medical Sciences (CISMed), University of Trento, Trento, Italy; 9 High-Profile Area of Health Sciences, University of Bremen, Bremen, Germany; 10 Institute of General Practice, Friedrich-Alexander-University Erlangen-Nürnberg, Erlangen, Germany; 11 Department of Family Medicine, College of Human Medicine, Michigan State University, East Lansing, Michigan, United States of America; University Hospital Cologne: Uniklinik Koln, GERMANY

## Abstract

**Objectives:**

To evaluate the feasibility of a novel point-of-care test (POCT) management strategy including phase contrast microscopy for bacteriuria and urinary dipsticks for erythrocytes to guide antibiotic prescribing in women with suspected uncomplicated urinary tract infection (uUTI) in general practice.

**Design:**

Pilot cluster randomised controlled trial in 20 general practices in Germany. Practices were assigned 1:1 to POCT-guided management or usual care. All urine samples were sent for urine culture. Follow-up over 28 days involved symptom diaries, telephone interviews, and medical record review.

**Outcomes:**

Primary outcomes were recruitment and retention rates. Secondary outcomes included total and inappropriate antibiotic use, symptom duration and burden, recurrent and upper UTIs, re-consultations, and diagnostic accuracy of microscopy versus urine culture. Mixed-effects models accounted for clustering.

**Results:**

Over 8 months, 157 women were recruited (90 intervention, 67 control), median of 7.5 patients per practice (range 1–15). Participant retention at day 28 was 75%. Baseline characteristics were well balanced. Antibiotic use was similar in both groups: 77% (intervention) vs. 79% (control) at initial consultation. The mean number of antibiotic courses over 28 days was 0.96 (intervention) vs. 1.00 (control), with no indication of reduced prescribing. Phase-contrast microscopy showed limited diagnostic accuracy, especially for ruling out infection (negative predictive value 46%). Exploratory analyses suggested that if GPs had access to urine culture results at the point of care, antibiotic prescribing in the intervention group could have been higher than in routine care.

**Conclusion:**

The POCT-guided management approach for suspected uUTIs is feasible but presents implementation and methodological challenges. Recruitment varied across sites and was lower in the control group practices, highlighting the risk of differential recruitment. Retention was below the expected 80%, indicating the need for efficient follow-up strategies in future trials. Explorative analyses suggest that simply adding diagnostic information may not support antibiotic stewardship. Novel POCTs should be carefully assessed for their influence on prescribing before routine use. **Trial registration**: ClinicalTrials.gov NCT05667207.

## Introduction

Despite being self-limiting in up to 50% of cases, most women with uncomplicated urinary tract infections (UTIs) receive antibiotics, as recommended by clinical guidelines [1–4]. This widespread use contributes to the rise of antibiotic-resistant microorganisms, which makes it urgent to reduce unnecessary prescriptions [5,6]. Randomised controlled trials (RCTs) have investigated alternatives to immediate antibiotic treatment such as delayed prescriptions, herbal remedies, and non-steroidal anti-inflammatory drugs. These strategies significantly reduced antibiotic use compared to standard care. However, they also increased the risk of prolonged symptoms, higher symptom burden, incomplete recovery, febrile UTI, pyelonephritis, and antibiotic use at follow-up [7–11].

A meta-analysis of RCTs with data from 3,524 participants confirmed these outcomes and highlighted that women with positive urine cultures and erythrocytes in urine were more likely to experience incomplete recovery without antibiotics. However, when both of these tests were negative there were no significant differences in terms of incomplete recovery between non-antibiotic strategies and immediate antibiotics. In addition, the study showed that positive urine cultures and erythrocytes in urine were predictive for the occurrence of complications and for the use of antibiotics during follow-up [12].

Urinary dipsticks can easily detect erythrocytes at the point of care, while standard urine cultures take over 48 hours, delaying treatment decisions. Studies on portable devices for rapid urine culture have shown limited effectiveness in supporting more appropriate treatment decisions [13–15], partly because they still require 24 hours for results and thus cannot inform immediate prescribing.

Urine microscopy, especially phase-contrast microscopy, is currently the only rapid POC test (POCT) evaluated in general practice for detecting bacteria in urine [16]. It is easier to implement than several other methods as it doesn’t require specimen preparation. Although reported sensitivity ranges from 74% to 95% and specificity from 63% to 97% [16], further evidence is needed to confirm the diagnostic accuracy of phase-contrast microscopy for UTIs in general practice and to understand its influence on antibiotic prescribing decisions.

To assist the planning of a confirmatory RCT, this pilot study evaluated the feasibility of a POCT-guided approach to reduce antibiotic use in women with suspected uncomplicated UTI (uUTI) in primary care. The POCTs were phase-contrast microscopy to detect bacteria and urinary dipstick to detect erythrocytes in urine specimens.

## Methods

### Design

The Microscopy in Urinary Tract Infection (MicUTI) trial was an open label primary care cluster pilot RCT comparing POCT-based management with usual care for women presenting in general practices with a suspected uUTI. The trial protocol was developed in accordance with the SPIRIT guidelines [17]. Below we summarise the methodology that was published in detail separately [18]. We followed the CONSORT guidelines for pilot [19] trials for reporting the results.

### Practice and patient recruitment

Twenty general practices from the Bavarian Practice-Based Research Network (BayFoNet) were randomised to the intervention or to the control arm in a 1:1 ratio. Over an eight-month period, GP practices executing the trial were asked to recruit and consent women aged 18–70 years with at least two out of four typical UTI symptoms (urgency, burning sensation, frequency, lower abdominal pain) and with no signs of a complicated infection (see S1 Table for the detailed criteria). Details of timing and trial procedures are visually summarised in S1 Fig. S1 Text provide details on incentives provided to practices and patients for participation in the trial.

### Randomisation

Minimisation was performed once after completing practice recruitment and obtaining their size ranges based on predefined thresholds (500−999, 1000−1499, 1500−1999, or ≥2000 patients per quarter). Practice sizes were approximated by the midpoint of the intervals. The minimisation involved two phases. First, 8 of 20 practices (40%) were randomised – 2 intervention and 2 control practices each pertaining to the two academic centres conducting the trial (University Hospital Würzburg and Erlangen). Second, the remaining 12 practices (60%) were allocated via constrained optimisation, ensuring an equal split between arms in both regions and minimising the between-arm difference in average practice size. The outcome of the minimisation (40% randomisation then 60% optimisation) was used as randomisation list.

The randomisation algorithm was programmed by the trial statistician (GB) and executed centrally by the statistical consultant (AH, who was not involved in trial management).

### Sample size

As this was a pilot trial, the primary objective was to assess feasibility parameters, not to evaluate the effectiveness of the intervention. In this context, performing a formal effectiveness-based sample size calculation would have been inappropriate and potentially misleading [19]. Instead, we aimed to enrol a sample sufficient to inform the design of a future definitive trial, guided by available routine data on UTI incidence and recruitment capacity in participating practices. We based our estimate of being able to recruit approximately 10 patients in each of the 20 practices over a six-month trial duration on an ad hoc analysis on claims data provided by the Bavarian Association of Statutory Health Insurance Physicians (Kassenärztliche Vereinigung Bayerns). This analysis [20], unpublished at the time of planning, identified around 2.2 million outpatient UTI cases between 2013 and 2019, corresponding to an average of more than 300,000 cases per year and indicating sufficient patient volume to meet the estimated recruitment target.

### Study arms

#### Intervention arm.

The intervention was based on the findings of a recent individual participant data meta-analysis of RCTs [12]. This study showed that in women treated without antibiotics, incomplete recovery was more likely if both erythrocytes in urine and urine culture were positive (Odds ratio [OR] 4.7, Bayesian credible interval (CI) 2.1 to 10.8). In contrast, no significant difference was detected in the likelihood of incomplete recovery between non-antibiotic strategies and immediate antibiotics when both tests were negative (OR 0.8, CI 0.3–2.0) [12].

Based on these findings, medical assistants in the intervention arm underwent a structured training in phase-contrast microscopy to detect bacteria in urine specimens prior to patient enrolment. A refresher training to ensure retention of skills was performed three months later in each intervention practice. Medical assistants were instructed to use phase-contrast microscopes (Primostar, Carl Zeiss, Suzhou, China) to examine 7 µL of clean-catch midstream urine without centrifugation at 400 × magnification in a precision counting chamber (Fast-Read 102 slides, Biosigma, Italy). A test was considered positive if more than a few bacteria of the same shape or many of different shapes were seen per high-power field [18]. Furthermore, practice teams were instructed to perform dipstick testing to detect erythrocytes in each patient’s urine. Microscopes, test strips (COMBUR5, Roche Diagnostics GmbH, Mannheim, Germany), and the necessary laboratory equipment were provided free of charge to participating general practices by the Department of General Practice, University Hospital Würzburg.

The results of the above POCTs were communicated to the consulting GPs, who were encouraged to apply the following management strategy:

1. If POCTs are positive for bacteria by microscopy and/or for erythrocytes by dipsticks, the GPs issue, at their discretion, a delayed or immediate prescription for an antibiotic.
2. If POCTs are negative for bacteria and erythrocytes, the GPs recommend no antibiotics and alternative treatments, such as nonsteroidal anti-inflammatory drugs, herbal formulations or delayed antibiotics according to current guidelines [4].

#### Control arm.

GP practices in the control arm performed usual care. In Germany, this usually consists of an antibiotic treatment based on symptoms and, optionally, dipstick testing [21].

### Outcomes

The primary outcomes were recruitment efficacy, calculated as the number of participants enrolled per site over the 6 and 8 months of trial duration, and retention rates, calculated as the percentage of complete follow-ups over 28 days.

Secondary outcomes included total antibiotic prescriptions (day 0–28), defined daily doses of prescribed antibiotics per patient (day 0–28), inappropriate antibiotic use (i.e., the antibiotics prescribed at initial consultation to women with a urine culture later confirmed as negative), number of antibiotic prescriptions at initial consultation (day 0), and diagnostic accuracy of POCTs compared to the reference standard of urine culture. Other secondary outcomes were also measured, mainly to explore the feasibility of these measures and to pilot data collection methods. These included number of early relapses (day 1–14) or recurrent UTIs (day 15–28), number of upper UTIs (day 1–28), time to symptom resolution, and total symptom burden on day 0–6 (area under the curve of the total symptom score). We originally planned to include the total symptom burden on day 0–7. However, because only 48% of patients reported data for this outcome up to day 7, we decided to reduce the number of days included to 0–6.

In addition to these prespecified outcomes, we measured the adherence to the intervention by calculating the proportion of initial consultations in which the GPs in the intervention arm did (or did not) prescribe antibiotics according to the algorithm’s recommendation. Moreover, we explored the following outcomes: 1) antibiotic prescribing at the initial consultation in consultations where the intervention was followed (per protocol population); and 2) antibiotics prescribed and antibiotics taken between days 0 and 14, with the latter according to data from patient diaries. Finally, we assessed how many antibiotics would have been prescribed during initial consultation if the urine culture results (the reference test) had been available and if the GPs had followed the recommended algorithm.

Results of the analyses of interviews conducted with practice teams to assess the acceptability of training sessions on POC tests will be published separately.

### Data collection

The practice teams were instructed to evaluate each woman who showed symptoms of an acute uUTI for possible inclusion. Following the provision of written informed consent, women who met the eligibility criteria filled out a validated questionnaire to rate the severity of their symptoms and the impact on their daily activities [22]. They also provided a urine sample for dipstick, culture, and, in intervention arm practices, microscopic analysis. Self-directed patient diaries based on the validated 8-item UTI symptoms and impairment questionnaire [22], telephone follow-up calls four weeks after inclusion, and electronic medical record (EMR) reviews were used to collect follow-up data until day 28 from inclusion. Telephone follow-up calls and EMR reviews were performed by study personnel from the involved academic departments (University Hospital Würzburg, University Hospital Erlangen, LMU University Hospital Munich) [18].

Urine cultures were performed for all patients by a single central laboratory based at the University of Würzburg. Details of urine specimen collection and analytical procedures are provided in the previously published protocol [18]. According to the updated 2020 German infectious disease and microbiology laboratory quality standards, culture results were considered positive if a typical uropathogen (e.g., *Escherichia coli* or *Klebsiella spp.*) was detected at ≥10³ CFU/mL or if a potential uropathogen (e.g., *Enterococcus spp.*) was found at ≥10³ CFU/mL with leukocytes present [23].

### Data analysis

We used appropriate descriptive statistics (e.g., means and standard deviations (SD) median and interquartile ranges (IQR), or ranges, frequencies and percentages) to summarise the characteristics of clusters and women.

For recruitment, which was measured as the number of patients per practice, the data exhibited overdispersion, necessitating the use of negative binomial regression. Retention, measured at an individual level as a binary outcome, was analysed using mixed-effects logistic regression to account for clustering and adjust for covariates.

Alongside point-estimates, we calculated 95% prediction intervals to estimate the expected range of recruitment and retention rates in future trials.

Secondary and exploratory outcomes were analysed using a range of statistical models tailored to the scaling of these variables. Binary outcomes were modelled using mixed-effects logistic regression, while continuous outcomes were analysed using mixed-effects linear models. For count data, mixed-effects Poisson models were employed. Time-to-event outcomes were analysed using mixed-effects Cox regression to appropriately account for censoring. For all models, we included the cluster (i.e., the GP practice) as a random effect and the arm and practice size as fixed effects. To assess the diagnostic accuracy of phase-contrast microscopy, we cross-tabulated microscopy test results by those of the urine culture (reference standard) and calculated point estimates and 95% CIs of diagnostic performance measures (sensitivity, specificity, likelihood ratios, predictive values). Adherence to algorithm recommendations was assessed via cross-tabulation and using the kappa statistic interpreted using standard cut-offs. Intraclass correlation coefficients (ICCs) were calculated for all secondary outcomes using the adjusted variance from mixed-effects models. We used R version 4.4.2 [24] and Stata version 18 [25] for all statistical analyses.

### Ethics

The Ethics Committee of the University of Würzburg in Germany approved the trial (reference number: 109/22-sc, Trial registration, ClinicalTrials.gov NCT05667207). All included women provided written informed consent for participation.

## Results

Twenty general practices affiliated with the Bavarian Practice Based Research Network (BayFoNet) were recruited (S2 Table shows practice level baseline characteristics) and randomised in May 2023. No practice dropped out of the study.

### Recruitment

Overall, patient enrolment lasted from June 23, 2023 (first patient in) to March 5, 2024 (last patient out: April 2, 2024). Over the initially planned 6-month trial duration per practice, 141 patients were enrolled in total (81 intervention, 61 control). The median number of recruited women per practice was 7.5 (9.0 intervention, 5.5 control) with a range of 1–14 (1–14 intervention, 1–12 control), which corresponds to a coefficient of variation (CV) of 58% (54% intervention, 62% control). As we did not reach the initially targeted sample size of ten patients per practice, practices were given extra two months of recruitment time. Over 8 months of trial duration, 157 patients were enrolled (90 intervention, 67 control) with a median number of recruited patients of 7.5 (10.0 intervention, 6.0 control), range 1–15 (1–15 intervention, 1–13 control), CV 57% (51% intervention, 63% control).

The model-based 95% prediction intervals provide ranges in which recruitment rates of future trials will lie with 95% probability, stratified by practice size. These predicted recruitment rates ranged from 0 to 12 in the smallest practices and 3–25 in the biggest practices in the intervention arm. In the control arm, the predicted recruitment ranged from 0 to 17 (smallest practices) and 1–19 (biggest practices, S3 Table), showing weaker dependence on practice size than in the intervention arm.

**Fig 1:**
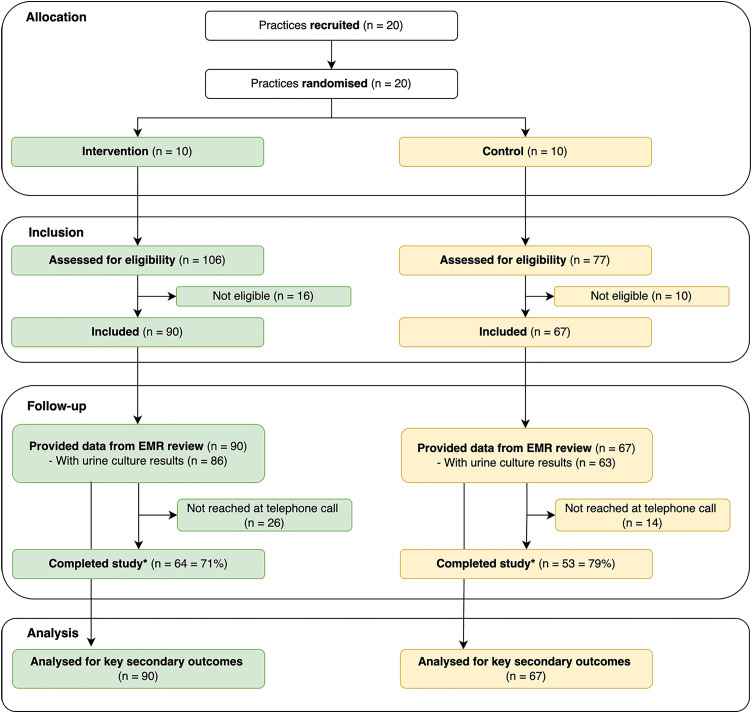
CONsolidated Standards of Reporting Trials (CONSORT) diagram showing the flow of cluster and participants through the trial. *A participant was defined to have completed the study if she was reached at the final telephone call. Abbreviations. EMR = Electronic medical record. Follow-up was 28 days.

Fig 1 shows the flow of clusters (practices) and participants through the trial.

### Patient level baseline characteristics

Women in the intervention group were slightly older (mean age 48.3 years, SD 13.7) than in the control group (mean age 43.1 years, SD 14.7). Across the 20 practices, patients in the two randomised arms were well balanced with respect to other baseline characteristics (Table 1). Overall, 68.5% of women had a positive urine culture (70% intervention, 67% control). The number of missing values per participant ranged from zero to six, being mostly three or fewer.

**Table 1:** Patient level baseline characteristics.

	Intervention (n = 90)	Control (n = 67)	Overall (n = 157)
**Variable**	**n/N (%) or mean (SD)**	**n/N (%) or mean (SD)**	**n/N (%) or mean (SD)**
**Mean (SD) age (years)**	48.3 (13.7)	43.1 (14.7)	46.1 (14.3)
**Symptoms at inclusion**			
Urgency	80/87 (92%)	65/66 (98%)	145/153 (95%)
Burning sensation	73/88 (83%)	53/66 (80%)	126/154 (82%)
Frequency	80/88 (91%)	61/66 (92%)	141/154 (92%)
Low abdominal pain	67/89 (75%)	53/66 (80%)	120/155 (77%)
**Symptom duration before consult ≤ 2 days**	41/86 (48%)	29/66 (44%)	82/152 (54%)
**Mean (SD) symptom sum score**	12.3 (3.5)	12.8 (3.0)	12.5 (3.3)
**Mean (SD) activity impairment sum score**	12.2 (3.7)	11.3 (3.4)	11.8 (3.6)
**Recurrence (at least 3 in previous year)**	28/89 (31%)	18/66 (27%)	46/155 (30%)
**Dipstick results**			
Erythrocytes positive	63/84 (75%)	50/62 (81%)	113/146 (77%)
Leukocytes positive	83/89 (93%)	62/66 (94%)	145/155 (94%)
Nitrites positive	22/85 (26%)	15/63 (24%)	37/148 (25%)
**Positive urine culture**	60/86 (70%)	42/63 (67%)	102/149 (69%)
*Escherichia coli*	46/60 (77%)	32/42 (76%)	78/102 (77%)
*Enterococcus faecalis*	20/60 (33%)	13/42 (31%)	33/102 (32%)
*Staphylococcus saprophyticus*	2/60 (3%)	4/42 (10%)	6/102 (6%)
*Klebsiella pneumoniae*	4/60 (7%)	1/42 (2%)	5/102 (5%)
*Proteus mirabilis*	1/60 (2%)	2/42 (5%)	3/102 (3%)
*Streptocococcus agalactiae*	2/60 (3%)	0 (0%)	2/102 (2%)
Other uropathogens	3/60 (5%)	3/42 (7%)	6/102 (6%)
**Contamination**	15/86 (17%)	16/63 (25%)	31/149 (21%)
**No growth**	5/86 (6%)	3/63 (5%)	8/149 (5%)

### Retention

By the end of the 28-day follow-up, 117 (75%) participants were reached by telephone, 137 (87%) returned their diary, but less than half (n = 76, 48%) completed it as intended. In the intervention arm, the corresponding figures were 64 (71%), 76 (84%), and 45 (50%), while in the control arm, they were 53 (79%), 61 (91%), and 31 (46%), respectively. S4 Table shows prediction intervals: as for recruitment, retention showed a weaker dependence on practice size in control arm practices than in the intervention arm. S5 Table shows the number of patients analysed for each secondary outcome.

### Secondary outcomes

Secondary outcomes are summarised in Table 2. Over the 28 days of follow-up, the mean number of antibiotic courses prescribed per patient was 0.96 in the intervention group and 1.00 in the control group, with an adjusted mean difference (aMD) of −0.06 (95% CI −0.38–0.25). Most antibiotics were prescribed at the initial consultation, with 77% of patients in the intervention group and 79% in the control group receiving a prescription (adjusted odds ratio (aOR) 1.01, 95% CI 0.16–6.38). With respect to appropriate antibiotic prescribing at initial consultation, 19 of 26 (73%) of intervention patients and 71% of controls received antibiotics despite negative culture results (aOR 1.22, 95% CI 0.23–6.39).

**Table 2:** Secondary outcomes.

	Intervention (N = 90)	Control (N = 67)	Missings n (%)	Overall (N = 157)	Intervention effect *b* or OR or HR (95% CI)	
*Outcome measure*			Intervention/ control		Crude	Adjusted
**Number of antibiotic courses Days 0–28**						
Mean (SD)	0.96 (0.58)	1.00 (0.60)	0/ 0	0.97 (0.59)	−0.04	−0.06 (−0.38–0.25)
Total n (%)	86 (96%)	65 (97%)				
During follow-up (day 1–28) n (%)	17 (20%)	14 (22%)				
**Number of antibiotic courses Day 0**						
Total n (%)	69 (77%)	53 (79%)	0/ 0	122 (78%)	0.87*	1.01* (0.16–6.38)
**Number of antibiotic courses Day 0 in patients with negative urine culture**						
Total n/N (%)	19/26 (73%)	15/21 (71%)	0/ 0	34/47 (72%)	1.08*	1.19* (0.24–5.83)
**Defined daily doses of antibiotics Days 0–28**	6.57 (8.91)	4.79 (6.59)	0/ 0	5.81 (8.03)	1.78	1.79 (−1.59–5.19)
**Number of early relapses (0–14)** According to EMR Review, Total n (%) According to telephone follow-up, Total n (%)	21 (23%) 8 (12%)	15 (22%) 5 (9%)	0/ 0 28 (31%)/ 16 (24%)	36 (23%) 13 (12%)	1.05* 1.36*	0.90* (0.26–3.10) 1.13* (0.25–5.13)
**Number of recurrent UTI (14–28)** According to EMR Review, Total n (%) According to telephone follow-up, Total n (%)	2 (2%) 2 (3%)	3 (5%) 3 (6%)	0/ 0 28 (31%)/ 16 (24%)	5 (3%) 5 (4%)	0.48* 0.53*	0.46* (0.07–2.88) 0.45* (0.07–2.89)
**Number of upper UTI** Total n (%)	4 (3%)	1 (2%)	0/ 0	5 (3%)	3.07*	2.91* (0.31–26.79)
**Number of consultations due to UTI** Mean (SD)	0.28 (0.47)	0.39 (0.72)	0/ 0	0.32 (0.59)	−0.11	−0.14 (−0.42–0.16)
**Time to symptom resolution** Mean days (SD)	6.05 (3.06)	5.33 (2.07)	33 (37%)/ 25 (30%)	5.75 (2.70)	0.64**	0.66** (0.43–1.01)
**Total symptom burden week 1** AUC Mean (SD)	60.12 (15.97)	58.63 (14.14)	25 (27%)/ 15 (22%)	59.47 (15.15)	1.5	1.18 (−4.51–6.86)

* Odds ratio from logistic regression model; ** Hazard ratio from Cox proportional hazard model.

Abbreviations: AUC = Area under the curve of the total symptom score, OR = odds ratio, HR = hazard ratio.

With regards to the outcomes number of consultations due to UTI, early relapses, recurrences, number of upper UTI, total symptom burden, and time to complete symptom resolution, no clear differences were observed for these outcomes. However, confidence intervals were wide, reflecting a high degree of uncertainty. For patient reported outcomes, the percentage of missing data was also high (22–37%).

Microscopy showed a sensitivity of 70% (95% CI 56–80) and specificity of 60% (95% CI 39–79) for detecting positive urine cultures, with a positive predictive value of 80% (95% CI 67–90) and a negative predictive value of 46% (95% CI 28–64). With a prevalence of culture confirmed UTI of 70%, the test identified 80% of culture-positive cases but had limited ability to rule out an infection (Table 3).

**Table 3:** Diagnostic accuracy of phase-contrast microscopy.

		Urine culture				
		**Positive**	**Negative**	Total		
**Microscopy**	**Positive**	41	10	51		
	**Negative**	18	15	33		
	Total	59	25	84		
**Prevalence, %** **(95% CI)**	**Sensitivity, %** **(95% CI)**	**Specificity, %** **(95% CI)**	**PPV, %** **(95% CI)**	**NPV, %** **(95% CI)**	**Positive LR (95% CI)**	**Negative LR (95% CI)**
70 (59–79)	70 (56–81)	60 (39–79)	80 (67–90)	46 (28–64)	1.74 (1.04–2.89)	0.51 (0.31–0.84)

Abbreviations. PPV = Positive predictive value, NPV = Negative predictive value, LR = likelihood ratio

*Urine culture positivity cut-off 10^3 CFU/ml

Algorithm adherence in the intervention arm (proportions of consultations in which the GPs followed algorithm recommendations) was 83%, compared to 69% expected by chance (kappa = 0.44, p < 0.0001, S6 Table). In the consultations in which the GPs adhered to the algorithm (per protocol population), antibiotic prescribing at the initial consultation was higher than in control arm (88% vs. 79%, aOR 2.51, 95% CI 0.32–19.98, S7 Table). In both groups, most prescriptions were issued within 14 days. During this period, the mean number of antibiotic courses per patient was 0.94 in the intervention group and 0.96 in the control group (aMD −0.03, 95% CI −0.33–0.27, S7 Table). The number of antibiotic courses actually taken was also similar between groups (0.83 vs. 0.85, aMD-0.02, 95% CI −0.30–0.26, S7 Table).

If urine culture results had been available and all GPs adhered to the study algorithm, 78 out of the 84 intervention patients with available data (93%) would have received antibiotics at the initial consultation. This compares to 53 out of 67 patients (79%) in the control group, resulting in an aOR for antibiotic prescribing of 3.77 (95% CI 0.86–16.52).

### Intra-cluster correlation coefficients of secondary outcome measures

To inform future cluster RCTs, we provide the intra-cluster correlation coefficients (ICCs) for secondary outcomes (S8 Table). ICCs ranged from 0.00 to 0.44, with the highest values observed for outcomes related to antibiotic use.

## Discussion

We evaluated the feasibility of a point-of-care test–guided management approach for suspected uUTIs in general practice compared to usual care. Although the intervention could be implemented in routine care, GP practices recruited fewer patients than initially expected. In line with previous primary care trials recruiting participants with an acute condition consecutively, the numbers of enrolled patients varied considerably across sites [7,8,15,26]. This variation reduces the effective sample size of a confirmatory cluster RCT, as unequal cluster sizes increase the design effect and diminish statistical power [27]. Fewer patients were enrolled in the control group (N = 67) than in the intervention group (N = 90). Recruitment tended to be higher in larger practices in the intervention group, but not in the control group. This, combined with the fact that control group practices recruited fewer patients than those in the intervention group, suggests that clinicians in control practices may have been less motivated to recruit participants. As a result, the influence of practice size on enrolment may have been less pronounced. Future studies should take this into account and, where possible, use individual randomisation or choose a cluster design where all sites can eventually implement the intervention (such as a cross-over or stepped wedge design) to reduce the risk of recruitment bias after randomisation.

Uneven recruitment patterns can also raise concerns about external validity [28]. Practices recruiting more patients may differ systematically from lower-volume or less-engaged practices – where clinical routines, diagnostic behaviours, or patient populations might not align with the broader population of GP practices.

Retention at day 28 was moderate, with 75% of participants reached by telephone and 87% returning symptom diaries, although less than half (48%) fully completed the diaries as instructed. This was lower compared to previous studies that used paper-pencil and telephone follow-ups [7,29], which achieved higher response rates at that time. The lower follow-up rates in the present study may reflect shifts in communication habits, with telephone calls and paper-pencil diaries becoming less effective. Electronic patient-reported outcome measures (ePROMs) using text message, emails or smartphone/web applications may offer a more convenient alternative by enabling real-time data collection and reducing the burden on participants [30,31].

With an OR of 1.01, we found no evidence that the trial intervention reduced antibiotic prescribing, either at the initial consultation or during follow-up. This could in part be attributed to the so-called Hawthorne effect, as participating GPs may have been more cautious or conservative with antibiotic prescriptions simply because they were aware of being part of a study [32]. This might explain why, at the initial consultation, antibiotics were prescribed to 122 (78%) women — a rate lower than reported in previous studies [15,33,34], but consistent with a recent trial conducted among 110 general practices in Germany [35]. The awareness of being monitored could have prompted GPs to exercise greater restraint when faced with diagnostic uncertainty, which may have diluted the potential impact of the intervention on prescribing behaviour. The intervention, however, did not appear to reduce antibiotic prescriptions in women with negative culture either, pointing towards the fact that the diagnostic strategy in the intervention group did not provide advantages in ruling out the infection, which is confirmed by our observation that phase-contrast microscopy performed poorly for ruling out the infection (negative predictive value = 46%). This adds to previous studies that have shown a very heterogeneous diagnostic performance of this microscopic method [36–39], with a systematic review concluding that evidence supporting its diagnostic accuracy in general practice was limited [16].

A more accurate diagnostic method, combined with better adherence than was actually observed, might have led to improved outcomes. Interestingly, exploratory analyses suggested that if urine culture results had been available at the point of care and all GPs had adhered to the algorithm, the estimated proportion of patients prescribed an antibiotic at the initial consultation would have been 93% in the intervention arm versus 79% in the usual care arm. This suggests a potential increase in antibiotic use rather than a reduction. Novel rapid point-of-care tests for UTIs are currently in development [40], with initial clinical evaluations underway [41]. However, our findings suggest that even if these tests prove to be accurate, they need to be thoroughly evaluated before entering routine clinical care. Diagnostic and management algorithms will need to be carefully developed and tested with patient-relevant health outcomes, as previously shown [42–45].

### Strengths and limitations

Strengths of the MicUTi trial include its rigorous cluster randomised design, the successful engagement of GP practices within a practice-based research network, and the comprehensive data collection ensuring near 100% completeness for the key secondary outcome of antibiotic prescription. The study implemented standardized POCT training for medical assistants and centralized urine culture testing, enhancing diagnostic reliability and comparability. Conducting the trial in routine primary care settings allowed for real-world assessment of the intervention feasibility. Additionally, the exploratory analyses provided insights into algorithm adherence and its implications for antibiotic prescribing, informing future research on diagnostic stewardship in primary care.

Several limitations must be considered. Recruitment rates varied considerably among practices and between both trial groups. This is a particular challenge in cluster trials for acute conditions (like UTIs), where patient enrolment depends on unpredictable presentation patterns, requiring a larger sample size to account for recruitment variability. In addition, women in the control group were younger (mean age 43.1) than those in the intervention group (mean age 48.3), indicating a potential for post-randomisation recruitment bias. This type of bias can occur when participants are recruited after randomisation and recruiters are aware of their cluster’s allocation. This was the case in our study, where allocation concealment or blinding were not possible, as clinicians were required to perform and interpret point-of-care tests as part of the intervention. To minimise such bias, future RCTs should, whenever possible, use individual randomisation, which prevents differential recruitment.

Despite the differences highlighted above, baseline characteristics were well balanced between study arms (thus, ensuring internal validity [28]), and the prevalence of culture-confirmed UTIs was 69%, suggesting that the study population was broadly representative of women seen in primary care for suspected uUTIs [12]. Selection bias may have occurred in practice recruitment, as participating practices were those interested in research, potentially leading to differences in prescribing behaviours or evidence-based decision-making compared to other practices. However, as most physicians prescribed an antibiotic at initial consultation, we do not expect this to have significantly impacted the results. Notably, we did not collect baseline data on antibiotic prescribing, which limits our ability to fully assess this potential bias.

Another limitation was that despite standardised training in phase-contrast microscopy, the method itself remains difficult to standardise in routine primary care. Microscopy results are highly dependent on the individual performing the test, and practice assistants may lack sufficient experience, especially in sites with low patient recruitment. This variability could have influenced diagnostic accuracy and, consequently, clinical decision-making.

Finally, while algorithm adherence among intervention GPs was moderate (83%), the study was not designed to assess the reasons for non-adherence, which may have influenced the observed antibiotic prescribing patterns.

## Conclusion

This trial showed that a point-of-care test–guided management approach for suspected uUTIs in general practice is feasible but presents challenges. First, uneven recruitment patterns across sites highlight the complexities of conducting cluster trials for acute conditions and raise concerns about external validity. Second, participant retention was limited—particularly for diary completion—indicating the need for efficient follow-up strategies in future RCTs, such as digital data collection tools or practice-level outcome measures. Finally, while novel POCTs are being developed, our findings suggest that simply providing additional diagnostic information may increase rather than reduce antibiotic prescribing if not carefully integrated into clinical decision-making. Their use must therefore be evaluated not only for diagnostic accuracy but also for their impact on prescribing behaviour in different patient populations.

## Supporting information

S1 Data Consort checklist. (DOC)

S1 Text Incentives provided to general practices and patients participating in the MicUTI RCT. (DOCX)

S1 Fig Cluster flow diagram – timeline cluster. (TIF)

S1 Table Inclusion and exclusion criteria. Abbreviations. UTI = urinary tract infection; eGFR = glomerular filtration rate.
(DOCX)

S2 Table Comparison of cluster characteristics in the two trial arms. Abbreviations. IQR = interquartile range. GPs = general practitioners.
(DOCX)

S3 Table Predicted recruitment rates (95% prediction intervals [PI]*). *Prediction intervals are based on two negative binomial regression models (one for each arm of the trial) using the practice size as a predictor.
(DOCX)

S4 Table Predicted retention rates (95% prediction intervals [PI]*). *Prediction intervals are based on two logistic mixed effects models (one for each arm of the trial) using the practice size as predictor and practice ID as random effect.
(DOCX)

S5 Table Participants analysed, by outcome. (DOCX)

S6 Table Fidelity to the algorithm´s recommendation in the intervention group. (DOCX)

S7 Table Antibiotic use in included women (exploratory outcomes). (DOCX)

S8 Table Intra-cluster correlation coefficients for secondary outcomes. (DOCX)
