# Supplementary material for: Dipsticks and point-of-care Microscopy in Urinary Tract Infections in primary care: Results of the MicUTI pilot cluster randomised controlled trial
Source: PLoS One. 2025 Oct 8;20(10):e0332390. doi: 10.1371/journal.pone.0332390 (PMC12507256; doi:10.1371/journal.pone.0332390)
Supplement: S1 Text — (DOCX) [file pone.0332390.s002.docx]

**S1 Text. Incentives provided to general practices and patients participating in the MicUTI RCT.**

General practices received the following incentives for participating in the study:

1. Research teams from two university departments (Department of General Practice, University Hospital Würzburg, and Department of General Practice, University Hospital Erlangen, Germany) visited each of the 20 participating general practices to present the study and provide instructions on patient recruitment, data collection, and documentation using paper-based case report forms (CRFs).
   - Physicians were compensated at a rate of 100 EUR per physician-hour for time spent during this visit.
   - Practice assistants were compensated at a rate of 20 EUR per person-hour.
2. In intervention practices, an additional focused training session (2–3 hours) was offered, primarily directed at practice assistants, to train them in the use of phase-contrast microscopy. For attending, practice assistants were compensated at the above hourly rate. Physician participation in this training was optional; if they attended, they were compensated at the same hourly rate as above.
3. For each patient recruited into the study, practices received a lump sum of 75 EUR.
4. Phase-contrast microscopes (in intervention practices only) and all necessary laboratory materials were provided free of charge by the Department of General Practice, University Hospital Würzburg, for the duration of the trial. Microscopes used in the study were purchased from Zeiss (Carl Zeiss, Suzhou, China).

Patients received a lump sum of 20 EUR upon completion of follow-up, defined as return of the patient diary and successful contact during the telephone follow-up call.
