# Supplementary figures and images for: Dipsticks and point-of-care Microscopy in Urinary Tract Infections in primary care: Results of the MicUTI pilot cluster randomised controlled trial
Source: PLoS One. 2025 Oct 8;20(10):e0332390. doi: 10.1371/journal.pone.0332390 (PMC12507256; doi:10.1371/journal.pone.0332390)

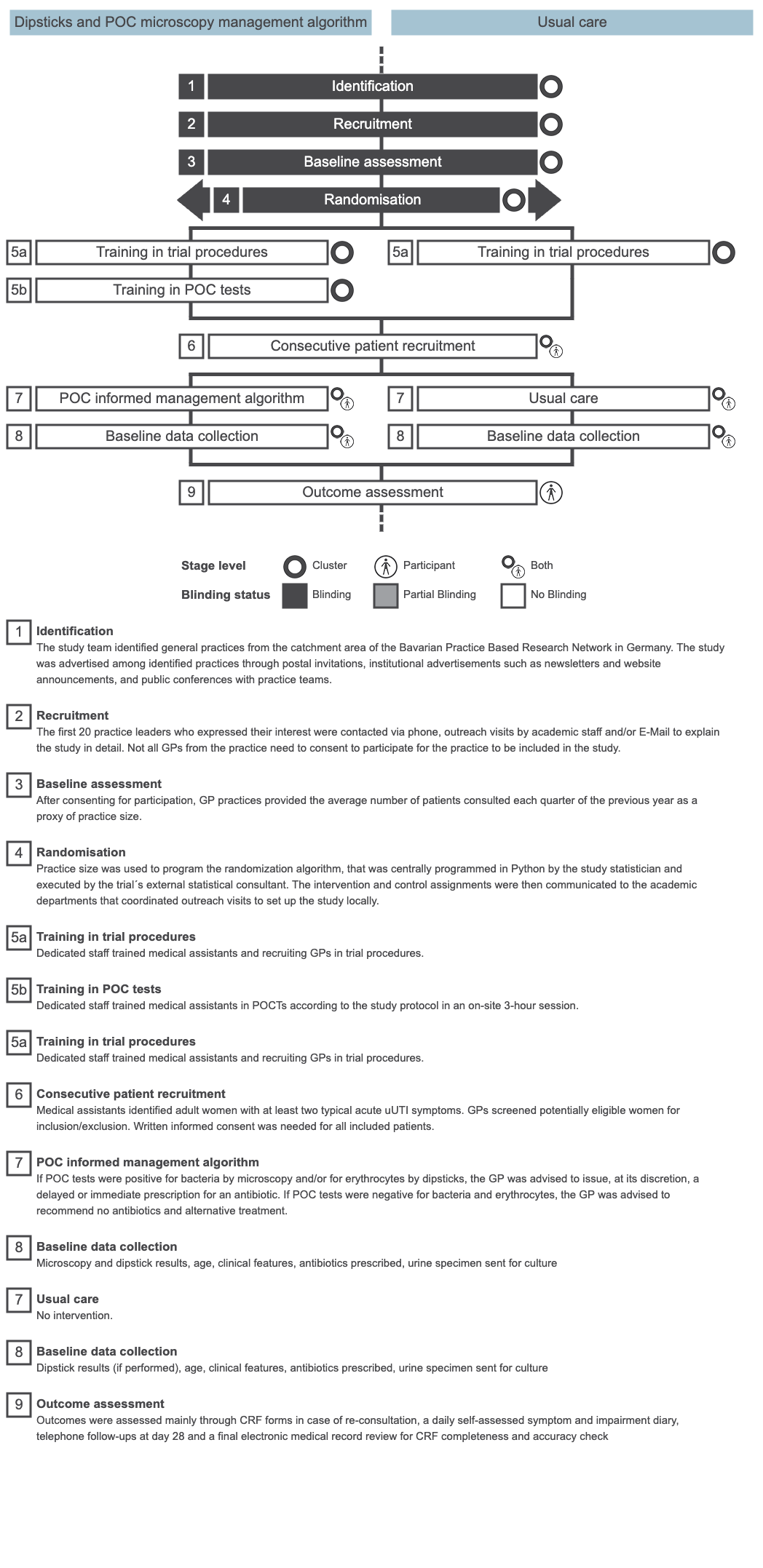

Supplement: S1 Fig — (TIF) [file pone.0332390.s003.tif]
