# Supplementary material for: Dipsticks and point-of-care Microscopy in Urinary Tract Infections in primary care: Results of the MicUTI pilot cluster randomised controlled trial
Source: PLoS One. 2025 Oct 8;20(10):e0332390. doi: 10.1371/journal.pone.0332390 (PMC12507256; doi:10.1371/journal.pone.0332390)
Supplement: S1 Table — Abbreviations. UTI = urinary tract infection; eGFR = glomerular filtration rate. (DOCX) [file pone.0332390.s004.docx]

**S1 Table**. **Inclusion and exclusion criteria.**

| **Inclusion criteria** |
| --- |
| Woman |
| Age 18-70 |
| At least two out of four typical UTI symptoms: dysuria, frequency, urgency, nocturia, lower abdominal pain |
| **Exclusion criteria** |
| Signs of a complicated UTI (history of fever, chills or flank pain). |
| Clinically relevant immunosuppression (i.e., current use of any immunosuppressive therapy, congenital or acquired disorders of immunity). |
| Acute or chronic functional or anatomical variations in the urinary tract except for chronic kidney failure with a glomerular filtration rate (eGFR) >45 mL/min. |
| Permanent bladder catheter or use of bladder catheter within the past 2 weeks. |
| UTI within the past 2 weeks. |
| Use of any antibiotic within the past 2 weeks. |
| Accommodation in a nursing home or hospital stay within the past 2 weeks. |
| Severe neurologic, psychiatric illness, severe dementia or severe substance use disorder. |
| Other severe diseases. |
| Being unable to understand the informed consent or to complete the patient diary. |
| Known pregnancy. |
