# Supplementary material for: Dipsticks and point-of-care Microscopy in Urinary Tract Infections in primary care: Results of the MicUTI pilot cluster randomised controlled trial
Source: PLoS One. 2025 Oct 8;20(10):e0332390. doi: 10.1371/journal.pone.0332390 (PMC12507256; doi:10.1371/journal.pone.0332390)
Supplement: S2 Table — Abbreviations. IQR = interquartile range. GPs = general practitioners. (DOCX) [file pone.0332390.s005.docx]

**S2 Table**. **Comparison of cluster characteristics in the two trial arms.**

|  | **Intervention** | | **Control** | |
| --- | --- | --- | --- | --- |
|  | n or median | % or IQR or range | n or median | % or IQR or range |
| **Practice size (median number of patients per quarter)** | 1750 | IQR 1250 - 2250 | 1750 | IQR 1250 – 2250 |
| **Number of recruiting GPs, per practice** | 1 | Range 1-2 | 1 | Range 1-1 |
| **Number of recruiting medical assistants, per practice** | 2 | Range 1-4 | 1 | Range 1-3 |
| Number of recruiting medical assistants, overall | 21 | N.A. | 15 | N.A. |
| **Number of rural practices (<5000 inhabitants)** | 5 | 50% | 6 | 60% |
