# Supplementary material for: Dipsticks and point-of-care Microscopy in Urinary Tract Infections in primary care: Results of the MicUTI pilot cluster randomised controlled trial
Source: PLoS One. 2025 Oct 8;20(10):e0332390. doi: 10.1371/journal.pone.0332390 (PMC12507256; doi:10.1371/journal.pone.0332390)
Supplement: S3 Table — *Prediction intervals are based on two negative binomial regression models (one for each arm of the trial) using the practice size as a predictor. (DOCX) [file pone.0332390.s006.docx]

**S3 Table. Predicted recruitment rates (95% prediction intervals [PI]*).**

|  | **Intervention** | | **Control** | |
| --- | --- | --- | --- | --- |
| Practice size (median number of patients per quarter) | 95% PI  lower limit | 95% PI  upper limit | 95% PI  lower limit | 95% PI  upper limit |
| 750 | 0 | 12 |  |  |
| 1250 | 1 | 14 | 0 | 17 |
| 1750 | 2 | 15 | 1 | 16 |
| 2250 | 2 | 19 | 1 | 16 |
| 2750 | 3 | 25 | 1 | 19 |
