# Supplementary material for: Dipsticks and point-of-care Microscopy in Urinary Tract Infections in primary care: Results of the MicUTI pilot cluster randomised controlled trial
Source: PLoS One. 2025 Oct 8;20(10):e0332390. doi: 10.1371/journal.pone.0332390 (PMC12507256; doi:10.1371/journal.pone.0332390)
Supplement: S4 Table — *Prediction intervals are based on two logistic mixed effects models (one for each arm of the trial) using the practice size as predictor and practice ID as random effect. (DOCX) [file pone.0332390.s007.docx]

**S4 Table. Predicted retention rates (95% prediction intervals [PI]*).**

|  | **Intervention** | | **Control** | |
| --- | --- | --- | --- | --- |
| Practice size (median number of patients per quarter) | 95% PI  lower limit | 95% PI  upper limit | 95% PI  lower limit | 95% PI  upper limit |
| 750 | 6.6% | 86.9% |  |  |
| 1250 | 13.3% | 90.6% | 31.3% | 96.4% |
| 1750 | 20.8% | 93.6% | 30.1% | 96.3% |
| 2250 | 33.0% | 96.5% | 32.5% | 97.1% |
| 2750 | 41.4% | 98.4% | 30.4% | 97% |
