# Supplementary material for: Dipsticks and point-of-care Microscopy in Urinary Tract Infections in primary care: Results of the MicUTI pilot cluster randomised controlled trial
Source: PLoS One. 2025 Oct 8;20(10):e0332390. doi: 10.1371/journal.pone.0332390 (PMC12507256; doi:10.1371/journal.pone.0332390)
Supplement: S5 Table — (DOCX) [file pone.0332390.s008.docx]

**S5 Table**. **Participants analysed, by outcome**.

|  | **Intervention (n=90)** | **Control (n=67)** |
| --- | --- | --- |
| **Outcome** | **n (%)** | **n (%)** |
| **Number of antibiotic courses Days 0-28** | 90 (100) | 67 (100) |
| **Number of antibiotic courses Day 0** | 90 (100) | 67 (100) |
| **Number of antibiotic courses Day 0 in patients with negative urine culture** | 86 (96) | 63 (94) |
| **Defined daily doses of antibiotics Days 0-28** | 90 (100) | 67 (100) |
| **Number of early relapses (0 – 14)** |  |  |
| According to EMR Review | 90 (100) | 67 (100) |
| According to telephone follow-up | 62 (69 | 51 (76) |
| **Number of recurrent UTI (14 – 28)** |  |  |
| According to EMR Review | 90 (100) | 67 (100) |
| According to telephone follow-up | 62 (69) | 51 (76) |
| **Number of upper UTI** | 90 (100) | 67 (100) |
| **Number of consultations due to UTI** | 90 (100) | 67 (100) |
| **Time to symptom resolution.** | 57 (63) | 42 (63) |
| **Total symptom burden week 1** | 66 (73) | 52 (78) |
