# Supplementary material for: Dipsticks and point-of-care Microscopy in Urinary Tract Infections in primary care: Results of the MicUTI pilot cluster randomised controlled trial
Source: PLoS One. 2025 Oct 8;20(10):e0332390. doi: 10.1371/journal.pone.0332390 (PMC12507256; doi:10.1371/journal.pone.0332390)
Supplement: S6 Table — (DOCX) [file pone.0332390.s009.docx]

**S6 Table. Fidelity to the algorithm´s recommendation in the intervention group.**

|  |  | **GP´s decisions at initial consultation** | | |
| --- | --- | --- | --- | --- |
|  |  | No Antibiotics | Antibiotics | Total |
| **Algorithm´s recommendation** | No antibiotics | 9 (64%) | 5 (36%) | 14 (16%) |
|  | Antibiotics | 10 (14%) | 64 (86%) | 74 (84%) |
|  | Total | 19 (22%) | 69 (78%) | 88 (100%) |
