# Supplementary material for: Dipsticks and point-of-care Microscopy in Urinary Tract Infections in primary care: Results of the MicUTI pilot cluster randomised controlled trial
Source: PLoS One. 2025 Oct 8;20(10):e0332390. doi: 10.1371/journal.pone.0332390 (PMC12507256; doi:10.1371/journal.pone.0332390)
Supplement: S7 Table — (DOCX) [file pone.0332390.s010.docx]

**S7 Table. Antibiotic use in included women (exploratory outcomes).**

|  | **Intervention (N = 90)** | **Control**  **(N = 67)** | **Missings**  **n (%)** | **Overall**  **(N = 157)** | **Intervention effect**  ***b* or OR (95% CI)** | |
| --- | --- | --- | --- | --- | --- | --- |
| *Outcome measure* |  |  | Intervention / control |  | Crude | Adjusted |
| **Number of antibiotic courses Days 0-14** |  |  |  |  |  |  |
| Mean (SD) | 0.94 (0.55) | 0.96 (0.53) | 0 / 0 | 0.95 (0.54) | -0.02 | -0.03 (-0.33–0.27) |
| Total n (%) | 85 (94%) | 64 (96%) |  |  |  |  |
| During follow-up n (%) | 15 (18%) | 11 (17%) |  |  |  |  |
| **Number of antibiotic courses taken Days 0-14** |  |  |  |  |  |  |
| Mean (SD) | 0.83 (0.55) | 0.85 (0.57) | 14 (16%) / 6 (9%) | 0.84 (0.56) | -0.02 | -0.02 (-0.30–0.26) |
| Total n (%) | 63 (83%) | 52 (85%) |  |  |  |  |
| **Number of antibiotic courses Day 0*** |  |  |  |  |  |  |
| Total n (%) | 64/73 (88%) | 53/67 (79%) | 2 (2%) / 0 | 117 (84%) | 1.88 | 2.51 (0.32–19.98) |

* The outcome *number of antibiotic courses Day 0* is calculated in the per protocol population, i.e. only including those consultations in which the GPs adhered to the study algorithm.
