# Supplementary material for: Dipsticks and point-of-care Microscopy in Urinary Tract Infections in primary care: Results of the MicUTI pilot cluster randomised controlled trial
Source: PLoS One. 2025 Oct 8;20(10):e0332390. doi: 10.1371/journal.pone.0332390 (PMC12507256; doi:10.1371/journal.pone.0332390)
Supplement: S8 Table — (DOCX) [file pone.0332390.s011.docx]

**S8 Table. Intra-cluster correlation coefficients for secondary outcomes.**

| **Outcome** | **Intra-cluster correlation coefficient** |
| --- | --- |
| Number of antibiotics, days 0 - 28 | 0,26 |
| Defined daily doses of antibiotics (DDDs), days 0 - 28 | 0,10 |
| Antibiotics in patients with negative urine culture | 0,44 |
| Number of antibiotics, days 0 | 0,35 |
| Number of early relapses, days 0 - 14 | 0,19 |
| Number of recurrent UTIs, days 15 - 28 | 0,00 |
| Number of upper UTIs, days 0 - 28 | 0,02 |
| Number of consultations due to UTI, days 0 - 28 | 0,15 |
| Time to complete symptom resolution | 0,00 |
| Total symptom burden, days 0 - 6 | 0,00 |
